# Hemocyanin from Shrimp *Litopenaeus vannamei* Has Antiproliferative Effect against HeLa Cell *In Vitro*

**DOI:** 10.1371/journal.pone.0151801

**Published:** 2016-03-23

**Authors:** Liyuan Zheng, Xianliang Zhao, Pei Zhang, Chuandao Chen, Shangjie Liu, Runqing Huang, Mingqi Zhong, Chiju Wei, Yueling Zhang

**Affiliations:** 1 Department of Biology and Guangdong Provincial Key Laboratory of Marine Biotechnology, Shantou University, Shantou, 515063, China; 2 Research Institute for Biomedical and Advanced Materials, Shantou University, Shantou, 515063, China; Stazione Zoologica Anton Dohrn, Naples, ITALY

## Abstract

Hemocyanin (HMC) has been shown to participate in multiple roles of immune defence. In this study, we investigated the antiproliferative effect and underpinning mechanism of HMC from *Litopenaeus vannamei in vitro*. Sulforhodamine B (SRB) assay indicated that HMC could dramatically inhibit the growth of HeLa cells, but not 293T cells under the same conditions. Moreover, typical morphological features of apoptosis in HeLa cells including the formation of apoptotic body-like vesicles, chromatin condensation and margination were observed by using 4, 6-diamidino-2- phenylindole dihydrochloride (DAPI) staining and fluorescence analysis. An apoptotic DNA ladder from 180 to 300 bp was also detected. Furthermore, 10 variation proteins associated with apoptosis pathway, viz. G3PDH isoforms 1/2 (G3PDH1/2), aldosereductase, ectodemal dysplasia receptor associated death receptor domain isoform CRA_a (EDARADD), heat shock 60kD protein 1 variant 1 (HSP60), heat shock 70kDa protein 5 precursor (HSP70), heat shock protein 90kDa beta member 1 precursor (HSP90), 14-3-3 protein ζ/δ, Ran and ubiquitin activating enzyme E1(UBE1), were identified from HMC-treated HeLa cells by the proteomic and quantitative real-time RT-PCR strategies. Importantly, the reactive oxygen species (ROS), mitochondrial membrane potential (Δψm) and caspase-9/3 activities were changed significantly in HMC-treated HeLa cells. Together, the data suggests that *L*. *vannamei* HMC mediates antiproliferative properties through the apoptosis mechanism involving the mitochondria triggered pathway.

## Introduction

Hemocyanin (HMC) is an extracellular giant copper-containing glycoprotein found in the hemolymph of both mollusk and arthropod. As the main protein component of hemolymph, it typically represents up to 95% of the total amount of protein. It is colorless in the deoxy form and blue in the oxygenous form, whose traditional function is responsible for oxygen transport [1]. Subsequently, it is demonstrated that HMC is also involved in several physiological processes, such as energy storage, osmoregulation, molt cycle and exoskeleton formation [2–4]. Later studies reveal that HMC can act as a multifunctional protein associated with the immune defense in invertebrates [5–6]. HMC from about 45 species, such as *Tachypleus tridentatus*, *Porcellio scaber*, *Cherax quadricarinatus*, and so on, could be functionally converted into a phenoloxidase-like enzyme [7–9]. Moreover, Zhang *et al*. reported that *Penaeus monodon* HMC could act as an antiviral agent against a variety of viruses including DNA and RNA viruses [10]. Zanjani *et al*. found *Haliotis rubra* HMC served as a new antiviral candidate for herpes simplex virus (HSV) infections [11]. Jiang *et al*. indicated that HMC from horseshoe crab *Carcinoscorpius rotundicauda* could possess a strong antimicrobial defense by the production of reactive oxygen species (ROS) activated with microbial proteases [12]. Destoumieux-Garzón reported that C-terminal fragments from HMCs in penaeid shrimps, *Penaeus vannamei* and *Penaeus stylirostris*, had broad antifungal activities [13]. Additionally, *Oncomelania hupensis* HMC could function as a vaccine in combination with Freund's adjuvant to evaluate the induction of immune responses and protection against *Schistosoma japonicum* infection in mice [14]. HMCs isolated from marine gastropods *Rapana thomasiana* and *Megathura crenulata* were doucumented to be acted as a potential bio-adjuvant for subunit vaccines [15]. Further, our previous evidence indicated that HMC from *Litopenaeus vannamei* could react with human IgG or IgA as an antigen [16, 17], bind to eight bacteria as an agglutinin [17], interact with inhomogeneous erythrocytes as a hemolysin [18], and enhance shrimp's immune response to immunostimulants as a related immune-enhancing protein [19].

Interestingly, accumulating evidences indicate that HMCs from some mollusks also have antitumor effects. For instances, in the early 1970s, Olsson *et al*. reported that keyhole limpet hemocyanin (KLH) strikingly decreased the tumor recurrence rate of patients with bladder transitional carcinoma cell (TCC) [20]. Lately, some researchers further illustrated that KLH could inhibit directly the growth of human breast and pancreatic cancer [21], barretts esophageal cancer [22], renal carcinoma [23], and prostate cancer cells *in vitro* [24]. Antonova *et al*. found HMCs derived from two snail species: *Helix lucorum* (HlH) and *Helix aspersa* (HaH) possessed antitumor effects on multiple malignant cell lines including bladder cancer (CAL-29 and T-24), ovarian cancer (FraWü), acute monocytic leukemia (THP-1), prostate cancer (DU-145), glioma cancer (LN-18), and Burkitt's lymphoma (Daudi) [25]. Gesheva *et al*. indicated that HMC from *Rapana thomasiana* (RtH) and *Helix pomatia* (HpH) expressed strong anti-cancer and anti-proliferative effects against colon carcinoma *in vivo* [26]. Arancibia *et al*. documented that HMC isolated from gastropod *Fissurella latimarginata* (FLH) or *Concholepas concholepas* hemocyanin (CCH) could act as an effective antiproliferative agent and decrease tumor growth [27, 28].

Notably, in contrast to the similarities in binding oxygen mollusk and arthropod HMCs are profoundly different in their molecular structure, size, and subunit organization. Generally, mollusk HMCs exist as decamers of several subunits with approximate masses of 350–450 kDa, each consists of 7 or 8 globular “functional units” connected by linker peptide strands, forming hollow cylindrical arrays with 5- or 10-fold axial symmetry. While arthropod HMCs are built on an entirely different plan, it consists of multiples of hexamers, each hexamer made of monomers of about 75 kDa. Because of these differences, it has now become customary to consider the mollusk and arthropod HMCs as different proteins [29–31]. However, so far little is known about the antitumor effects of HMC in arthropod.

In this study, the antiproliferative properties of HMC from shrimp *L*. *vannamei* against HeLa cells *in vitro* were investigated. Furthermore, the underlying mechanism was investigated via cellular, proteomics and molecular biology strategies. Our data will assist in the investigation of multifunctionality of HMC and help to establish a potential strategy for cancer control.

## Materials and Methods

### Animal and preparation of shrimp sera

Penaeid shrimps (*L*.*vannamei*), weighing about 18g, were obtained from Shantou Huaxun Aquatic Product Corporation (Shantou, Guangdong, China). They were cultured in tanks filled with 25 L of open-circuit filtered seawater at room temperature with free access to regular food and enough oxygen. Shrimps were acclimatized to laboratory conditions for 2 d before the experiments. Hemolymph was drawn directly from *L*.*vannamei* pericardial sinus and sera were separated as our previous descriptions [17]. The study protocol was approved by the Institutional Animal Care and Use Committee of Shantou University.

### Purification and identification of HMC

HMC purification was performed by affinity chromatography as described previously with modifications [18]. Briefly, a affinity chromatography column with a ligand of rabbit anti-shrimp HMC antibodies was installed according to the conventional method. After loading with *L*. *vannamei* sera (200 μL), the column was washed with PBS (0.01 M, pH 7.4) until the absorbance at 280 nm reached baseline. Bound HMC was eluted with glycine-HCl buffer (0.1 M, pH 2.4), and neutralized immediately with Tris-HCl buffer (1M, pH 8.0). After being concentrated, the total protein concentration was determined by the Bicinchoninic Acid assay (Genstar, China), and then stored in 0.01M pH7.4 PBS at -20°C until further identification and analysis.

Purified HMC was identified by gel electrophoresis and immunoblotting assays. SDS polyacrylamide gel electrophoresis (SDS-PAGE) was carried out under reducing conditions on a 10% polyacrylamide separating gel with a 5% stacking gel. The gel was stained with Coomassie Brilliant Blue R-250. For Western blotting, the proteins were transferred to a polyvinylidene fluoride (PVDF) membrane after SDS-PAGE with a semi-dry transfer apparatus according to the manufacturer's instructions. The membrane was blocked for 1 h with 5% skim milk in TBS (20 mM Tris, 0.15 M NaCl, pH 7.4) at room temperature, then incubated with rabbit anti-shrimp HMC antisera (1:1500 dilution) and goat anti-rabbit IgG-HRP (1:3000 dilution) antibodies at room temperature for 1 h and 40 min, respectively. Finally, the membrane was washed and developed with substrate (3'3-diminobenzidine, DAB) until optimum color was observed. In addition, bovine serum albumin (BSA) was used as a negative control to exclude potential non-specific reaction errors.

### Cell culture

Cervical carcinoma line HeLa cells were provided by Research Institute for Biomedical and Advanced Materials, Shantou University, Shantou 515063, China. Human embryonic kidney 293T cells, as a negative control, were given by Medical College, Shantou University, Shantou 515063, China. The cells were maintained in Dulbecco's Modification of Eagle's Medium (DMEM, Thermo, USA) supplemented with 10% fetal bovine serum (Gibco, USA), 100 IU/mL penicillin and 100 μg/mL streptomycin. Cells were cultivated in a humidified incubator containing 5% CO_2_ at 37°C.

### Sulforhodamine B (SRB) assay

The SRB assay was performed according to the report by Sagias *et al* [32]. In brief, HeLa or 293T cells were planted into a 96-well plate (5×10^3^/well) overnight. The cells were then treated with 10, 20, 30, 40 or 50μg/mL of HMC. PBS (0.01M pH 7.4) was used as a negative control, while 5-fluorouracil (5-FC, 20 μg/mL) was used as a positive control. After 24h or 48 h treatment, the supernatant was discarded, each well was then fixed with 50 μL of cold trichloroacetic acid (500 mg/mL) at 4°C for 1 h. After washing four times with deionized water, cells were incubated with 100 μL /well of SRB (4 mg/mL) for 30 min. Finally, the cells were washed with 1% acetic acid to remove excess dye, and then processed to solubilize the bound protein dye by adding 200 μL/well of 10 mM unbuffered Tris base. The absorbance at 515 nm was measured with a microplate reader (BioTek, USA). The inhibition rate (%) = (OD_0h_/OD_24h or 48h_-1) × 100%. All samples were prepared in triplicate and data are expressed as means ± standard error (SD). The *p* values were determined using Student's t-test.

### Cytological effects of HMC on HeLa cells

Following SRB analysis, the HeLa cells and 293T cells treated with PBS (0.01 M, pH 7.4), HMC (50 μg/mL) and 5-FC (20 μg/mL) for 48 h, were observed by an inverted microscope, respectively.

At the same time, the nuclear staining of HeLa cells by 4,6-diamidino- 2-phenylindole dihydrochloride (DAPI) was carried out as previously described [33]. Briefly, HeLa cells (5×10^3^/well) were treated with 10–50 μg/mL of HMC for 48 h. The cells were washed with PBS (0.01M, pH 7.4) twice and fixed with 2.5% glutaraldehyde for 20 min, and then washed two times with PBS (0.01M, pH 7.4). The fixed cells were stained with 1 μg/mL of DAPI for 10 min at room temperature. Cells were examined under a fluorescence microscope (Olympus, Tokyo, Japan) equipped with a CCD camera.

### Cell immunofluorescence assay

HeLa cells in log phase were harvested and incubated with 30 μL 3.5mg/mL HMC into immunofluorescence chamber at 37°C for 48 h. After washing with 0.01M pH7.4 PBS for twice, followed by 1% BSA for 30 min, then the cells were probed with rabbit anti-shrimp HMC (dilution 1:500) overnight at 4°C. After washing with PBS for three times, added cy3-conjugated anti-rabbit IgG (dilution 1:150) for 1 h. Then the nucleus is properly stained with 100 μL DAPI for 1–2 min, the antibody staining was visualized and images were obtained using an upright fluorescence microscope (Eclipse 90i, Nikon) under ×60 magnification.

### DNA ladder assay

DNA ladder assay was carried out as previously described [34]. HeLa cells (1 mL), treated with PBS (0.01 M, pH 7.4), HMC (50 μg/ml) and 5-FC (20 μg/ml) for 48 h, were fixed with 10 mL of 70% ethanol diluted in hanks´buffered salt solution (HBSS) for 24–72 h at room temperature. Cells were collected by centrifugation at 800 g for 5 min, and resuspended in 200 μL of 0.2 M phosphate-citrate (PC) buffer at room temperature for more than 30 min. After centrifugation at 1,000 g for 5 min, the supernatant was dried in a vacuum oven for 15 min. The sample was then incubated with 3 μL of 0.25% Nonidet NP-40 (FLUKA, U.S.A) and 3 μL of 1 mg/mL RNase A (Sigma, U.S.A) for 30 min, and 3 μL of 1 mg/mL proteinase K (Sigma, U.S.A) for an additional 30 min at 37°C. The DNA was eluted by addition of 70μL elution buffer (10 mM Tris-HCl, 0.1 mM EDTA; pH 8.8) followed by centrifugation at 13,000g for 1 min. After extraction and precipitatation, DNA was analyzed by electrophoresis in a 5% polyacrylamide gel at 140 V for 1–2 h. After rinsing with distilled water twice, the gel was stained with 0.2% aqueous ethanolic silver nitrate for 10 min. Finally, the gel was destained in distilled water until optimum DNA bands were observed.

### Two-dimensional gel electrophoresis (2-DE)

2-DE was performed as our previously described with modification [19, 35]. HeLa cells (5×10^3^/well) in 6-well plate were treated with 30 μg/mL of HMC or 0.01M pH7.4 PBS for 48 h. Whole cell proteins (300 μg) in rehydration buffer (containing 7 M urea, 2 M thiourea, 4% CHAPS, 65 mM DTT and 3.4 mL of IPG buffer, pH 3–10) was used to rehydrate the immobilized pH gradient (IPG) strip (7 cm, pH 3–10, Bio-Rad, USA) for 13 h. The isoelectric focusing (IEF) was performed at 20°C using a continuous increase in voltage (up to 6000 V) until reaching 42,000 Vh. And the following procedures including SDS-PAGE were same as described in our publication [19].

### Imaging and mass spectrometry analysis

Following 2-DE analysis, the gel images and differentially expressed proteins between the experimental group and control group were further analyzed with PDQuest software version 8.0 (Bio-Rad, Hercules, CA) and MALDI-TOF-TOF/MS, respectively. The detailed procedures were same as our previous descriptions [35].

### Quantitative real-time RT-PCR analysis

For further verification of the above results from mass spectrometry analysis, ten identified genes, viz. *G3PDH1*, *G3PDH2*, *aldosereductase*, *EDARADD*, *HSP60*, *HSP70*, *HSP90*, *14-3-3 protein ζ/δ*, *Ran* and *UBE1*, were amplified by using quantitative real-time RT-PCR assay. Total RNA was extracted from HeLa cells by using Trizol Reagent (Invitrogen, U.S.A). 5 μg of total RNA was reverse transcribed to cDNA using the PrimeScrip RT reagent Kit (TaKaRa, Dalian, China) following the manufacturer's instructions. The specific primers of target genes are listed in Table 1. The PCR procedures include 94°C for 2 min and 30 cycles of 94°C for 30 s and 58°C for 30 s and 72°C for 1 min, then a final elongation at 72°C for 10 min. As an internal loading control, beta-actin transcripts were amplified using the same amplification conditions. Data from the quantitative real-time RT-PCR analysis were subjected to the one-way analysis (one-way ANOVA) followed by an unpaired, two-tailed t-test. The fold change was calculated by the formula of 2^-ΔΔCt^, the mean of ΔΔCt was [(Ct._target gene_-Ct. beta-actin)_treated_—(Ct._target gene_—Ct. beta-actin)_control_ [35, 36]. All samples were prepared in triplicate, each including three technical replicates, and data are expressed as means±standard error (SD). Significant *p*-values were <0.05 or 0.01 on 2-tailed analysis.

**Table 1:** Nucleotide primers used for RT-PCR of differentially expressed proteins in HeLa cell treated with *Litopenaeus vannamei* HMC.

Primer	Sequence (5'-3')
β-actin-F	GTTGCGTTACACCCTTTC
β-actin-R	CTGTCACCTTCACCGTTC
G3PDH1-F	ACAACTTTGGTATCGTGGAAGG
G3PDH1-R	GCCATCACGCCACAGTTTC
G3PDH2-F	ACAACTTTGGTATCGTGGAAGG
G3PDH2-R	GCCATCACGCCACAGTTTC
aldosereductase-F	GTATTCTTTGGCAGGTTGTGGC
aldosereductase-R	AGGGAGGGCTGAAGTGGTGA
EDARADD-F	GTATTCTTTGGCAGGTTGTGGC
EDARADD-R	AGGGAGGGCTGAAGTGGTGA
HSP 60-F	CCTCATCTCACTCGGGCTTAT
HSP 60-R	CACCATCTTTTGTTACTTTGGGACT
HSP 70-F	TCCTATGTCGCCTTCACTCC
HSP 70-R	GCACAGACGGGTCATTCCAC
14-3-3 zeta/delta-F	AAGCCATTGCTGAACTTGATAC
14-3-3 zeta/delta-R	TTTTCCCCTCCTTCTCCTG
HSP 90-F	GCCAGTTTGGTGTCGGTTT
HSP 90-R	AGATGTGCTGGGTATCGTTGT
Ran-F	CCCTTCCTCTGGCTTGCTA
Ran-R	AGTCGTGCTCATACTGTGCTG
UBE1-F	TCGCCGCTGTCCAAGAAAC
UBE1-R	AGTAAAGGCCCTCGTCTATGTC

### Reactive oxygen species (ROS) measurement

ROS analysis of HeLa cells treated with HMC was carried out as previously described [37]. In brief, HeLa cells were planted at a density of 5×10^3^/well in a 96-well plate. One day after seeding, the culture dishes were treated with HMC (20–100 μg/mL), 0.01 M pH 7.4 PBS for 48 h, or positive drug Rosup (Beyotime, Haimen, China) at 37°C for 20 min. The treated cells were washed with PBS (0.01 M, pH7.4) twice and then directly treated with 10 μmol/L 2', 7' dichlorofluorescein diacetate (DCFH-DA) (Beyotime, Haimen, China) dissolved in serum-free medium at 37°C for 20 min. After washing with 0.01 M pH 7.4 PBS, ROS production in HeLa cells was detected by fluorescent signal with excitation and emission setting at 488 nm and 525 nm, respectively.

### Mitochondrial membrane potential assay

Mitochondrial membrane potential (Δψm) in HeLa cells was measured using the Tetrechloro-tetraethylbenzimidazol carbocyanine iodide (JC-1) mitochondrial membrane potential assay kit (Beyotime, Haimen, China). Briefly, HeLa cells were exposed to different concentration of HMC (20–100 μg/mL) for 48 h, or 10 μM carbonylcyanide-m-chlorophenylhy drazone (CCCP) as a negative control for 20 min. After washing with 0.01 M pH 7.4 PBS at least one time, the cells were incubated with JC-1 staining solution at 37°C for 20 min according to manufacturer instructions. Finally, both JC-1 monomers (λex 514 nm, λem 529 nm) and aggregates (λex 585 nm, λem 590 nm) were measured with a GloMax-Multi Detection System (Promega, America).

### Caspase-3/9 activity assay

Caspase-3/9 activity was assessed by using the caspase-3/9 cativity kit (Beyotime, Haimen, China). Briefly, HeLa cells in 6-well plates were pretreated with 20–60 μg/mL HMC for 48 h. The cells were harvested and lyzed on ice for 15 min. After centrifugation at 16,000 g at 4°C for 15 min, the supernatants were collected and protein concentrations were measured by Bicinchoninc acid (BCA) assay (Pierce, Rockford, IL). The cellular extracts (10 μL) were then cultured for 4 h with 80 μL reaction buffer containing 10 μL of caspase-3 substrate (Ac-DEVD-*p*NA) (2 mM) or caspase-9 substrate (Ac-LEHD-*p*NA) at 37°C. The absorbance of *p*NA, which represents the activity of caspase-3/9, was measured with a Multiscan Spectrum spectrophotometer (BioTek, USA) at 405 nm.

## Results

### HMC possessed antiproliferative effect aganist HeLa cells *in vitro*

**Fig 1:**
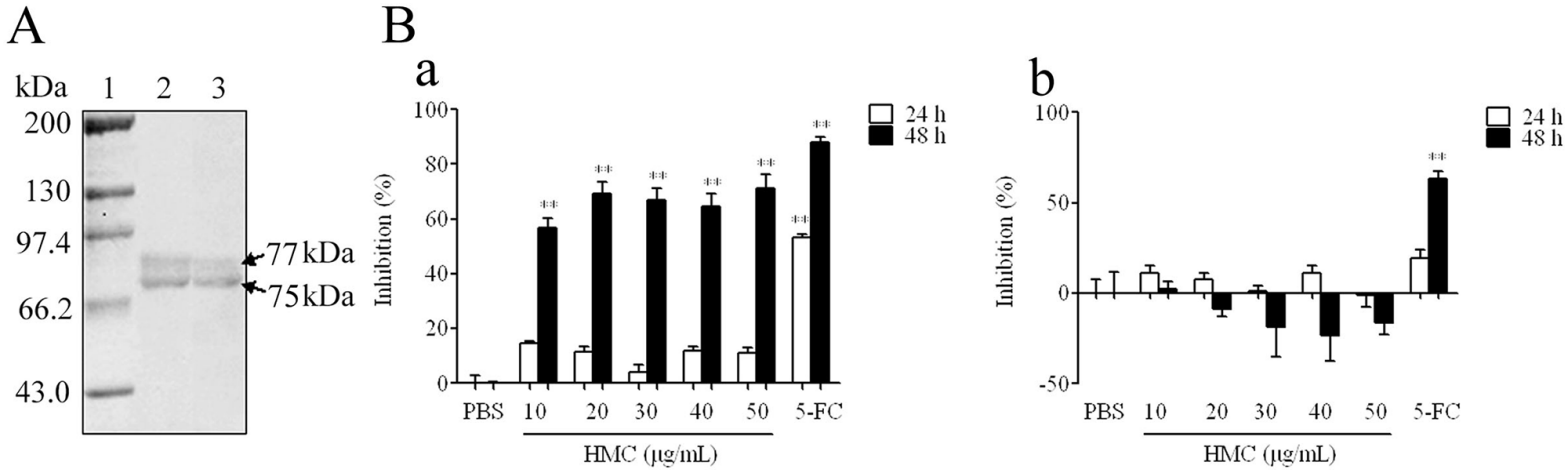
Identification and antiproliferative effect of *Litopenaeus vannamei* HMC. (A) SDS-PAGE and Western-blotting analysis. 1: Molecular mass markers; 2–3: SDS-PAGE and Western blotting analysis of HMC, respectively. (B) Inhibitory effects on cell growth of HMC. (a) HeLa cells, or (b) 293T cells were grown for 24h or 48 h in the presence of 10–50 μg/mL of HMC. Cell growth was analyzed by using the SRB assay. PBS was used as the negative control, while 5-FC was used as the positive control. Bars represent means±SD (n = 3). ** indicates significant difference between the HMC- (or 5-FC-) and PBS-treated cells (*p*<0.01).

HMC from the haemolymph of *L*. *vannamei* was purified by affinity chromatography as shown in Fig 1A. In consistent with our previous findings [18], two bands at molecular weights around 75 and 77 kDa were observed by SDS-PAGE, which could react with anti-shrimp HMC antibody specifically. Then, SRB assay was used to characterize the antiproliferative effect of HMC on HeLa cells viability. As shown in Fig 1B, an obvious inhibitory effect (57–71%) was observed in HeLa cells treated with 10–50 μg/mL of HMC for 48 h as compared to the untreated groups (*p*<0.01). In contrast, there was no significant difference in growth of 293T cells between the experimental group and negative group under the same conditions. In addition, a similar result was also found by using MTT analysis (data not shown). Thus, these data suggested that shrimp HMC exhibited antiproliferative effect on HeLa cells *in vitro* as an antitumor agent.

### Apoptosis was induced in HMC-treated HeLa cells

**Fig 2:**
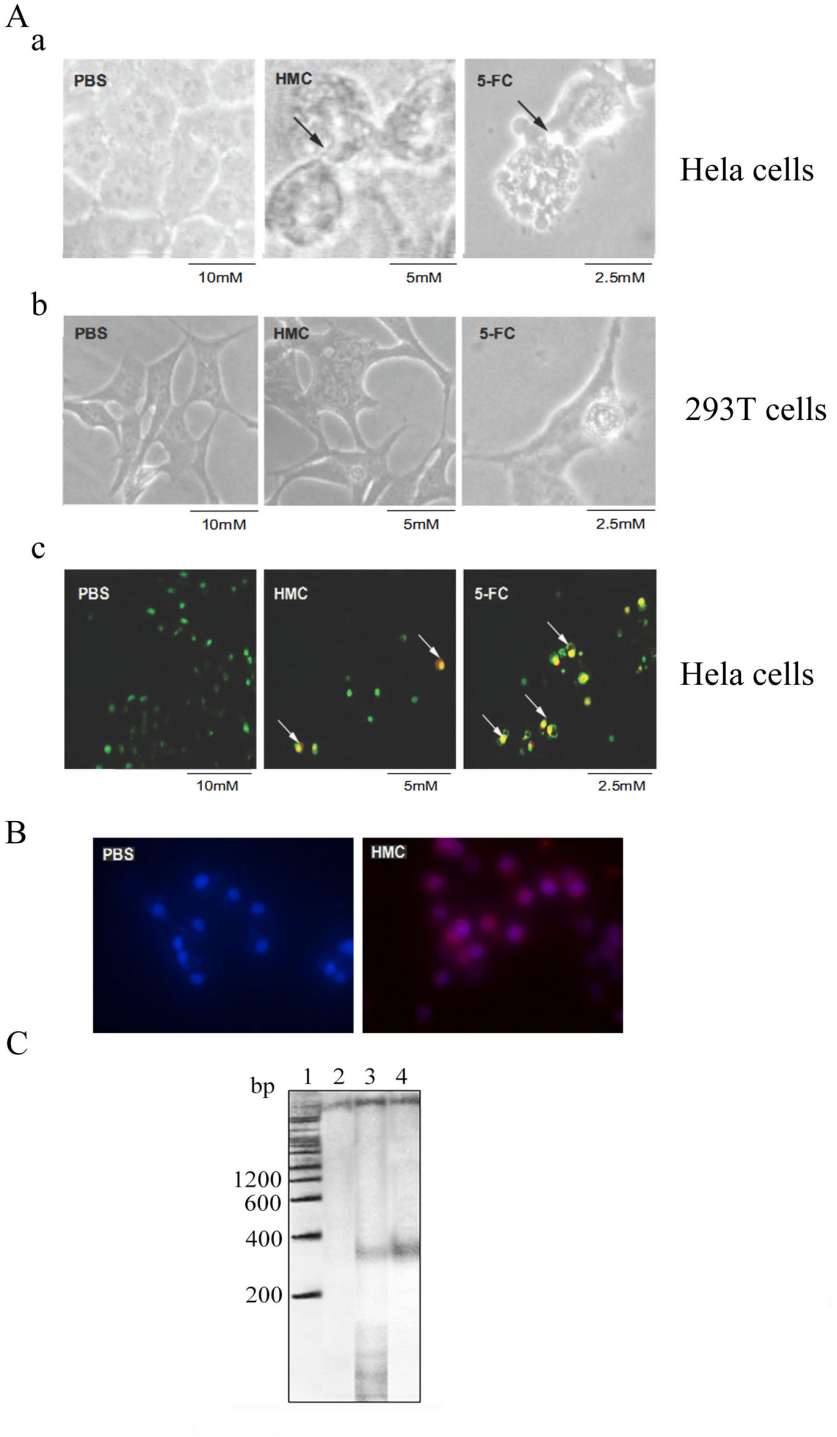
Antiproliferative effect analysis of *Litopenaeus vannamei* HMC at the cellular level. (A) Cytological effects. (a, b) Morphology changes of HeLa cells (a) and 293T cells (b) treated with PBS, HMC and 5-FC for 48 h. Micrographs were obtained from an inverted microscope (400×). The membrane blebbing indicated by arrows; (c) Nucleolus morphological changes of HeLa cells treatment with PBS, HMC and 5-FC for 48 h using DAPI staining observed under a fluorescence microscope (200×). The arrows point to the apoptotic body in HeLa cells. (B) Analysis of the interaction between Hela cells and HMC or PBS by cell immunofluorescence assay. (C) DNA ladder analysis. 1: Molecular mass markers; 2–4: HeLa cells treated with PBS, HMC and 5-FC, respectively.

To investigate the underlying mechanism of the antiproliferative effect of HMC at the cellular level, cell morphology, chromatin formation and immunofluorescence assay were determined. As shown in Fig 2A, HeLa cells were found to shrink and round up after 48 h exposure to HMC, accompanying with the appearance of membrane blebbing. Similar observations were found in 5-FC treated control, but not in PBS treated HeLa cells. Moreover, chromatin condensation, DNA fragmentation, chromatin margination and apoptotic body-like vesicles were revealed by DAPI staining in HMC-treated and 5-FC-treated HeLa cells but without the same phenomenon in PBS-treated group (Fig 2A). Notably, HMC (red) that distributed on the surface of HeLa cells (blue) by immunofluorescence assay, indicating that the interaction between HMC and HeLa cells (Fig 2B). In addition, a representative apoptotic DNA ladder around 180 to 300 bp was also observed in HeLa cells treated with HMC (50 μg/mL) for 48 h (Fig 2C). Thus, these results indicated that apoptosis was induced in HeLa cells treated with HMC.

### Identification of altered proteins in HeLa cells treated with HMC

**Fig 3:**
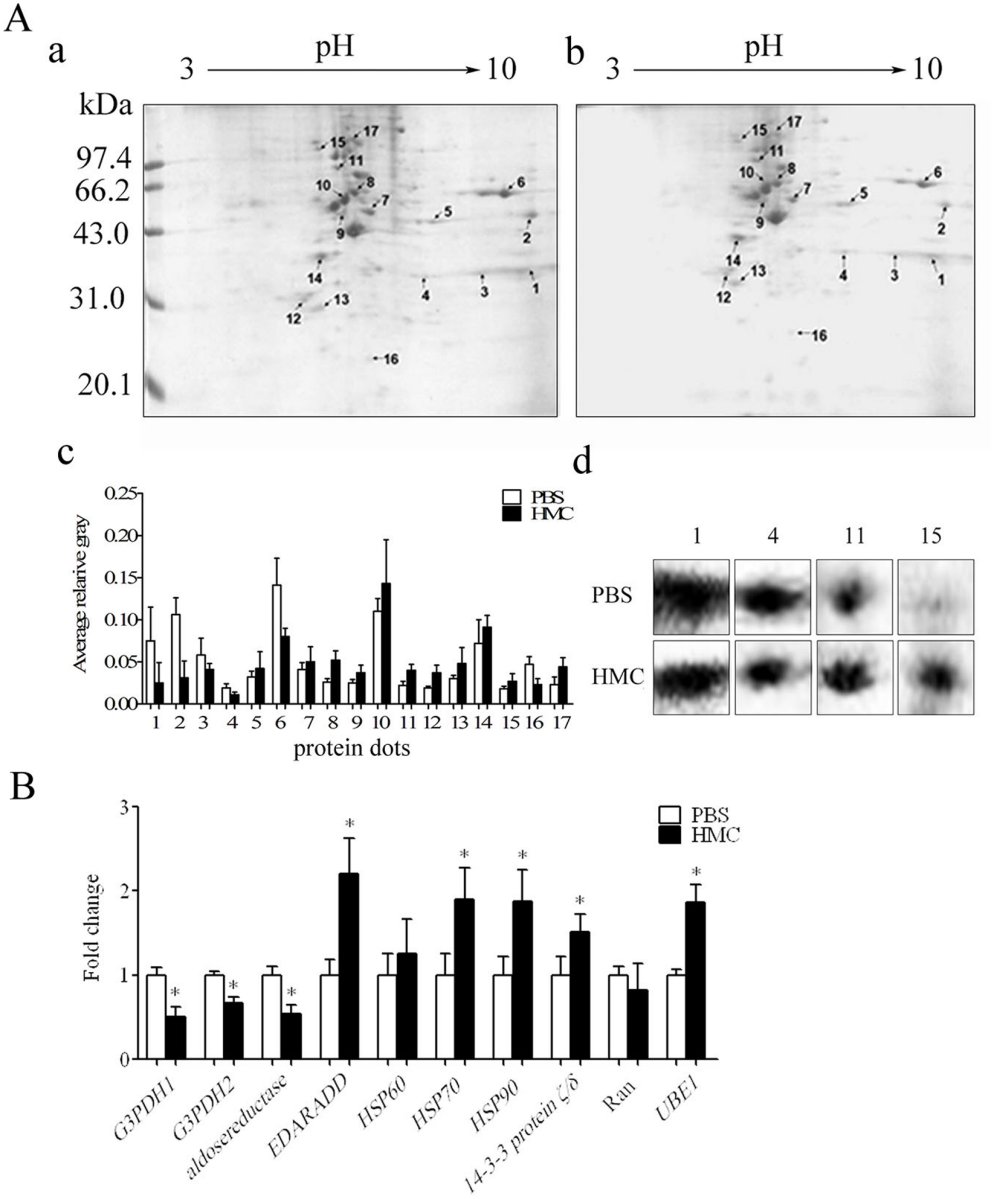
Identification of altered proteins in HeLa cells treated with *Litopenaeus vannamei* HMC. (A) 2-DE protein profiles. 2-DE analysis of total protein extracted from HeLa cells treated with PBS (a) and HMC (b) for 48 h; (c) Bar graph shows the gray value of differentially expressed proteins. Bars represent mean ± SD of normalized spot intensity determined according to PDQuest software version 8.0 (n = 3); (d) High light of four differentially expressed protein spots (spot 1, 4, 11 and 15). (B) Quantitative real-time RT-PCR analysis of *G3PDH1*, *G3PDH2*, *aldosereductase*, *EDARADD*, *HSP60*, *HSP70*, *14-3-3 protein ζ/δ*, *HSP90*, *Ran* and *UBE1* transcripts in HeLa cells after treatment with HMC for 48 h. Bars represent mean ±SD (n = 3). The significance difference between the HMC- and PBS-treated groups at 48 h was indicated with one asterisks (*p* < 0.05).

2-DE was performed to identify altered proteins of HeLa cells after stimulation with HMC for 48 h. Approximately, 17 altered protein spots with at least 1.5-fold difference compared with the control group were observed on the 2-DE gels (Fig 3A). Among them, 11 spots (5, 7, 8, 9, 10, 11, 12, 13, 14, 15, 17) were up-regulated and 6 spots (1, 2, 3, 4, 6, 16) were down-regulated. All differential spots were subjected to mass spectrometry analysis and 10 uniquely expressed proteins were identified (Table 2), which were G3PDH isoform 1 (G3PDH1, spot 1), G3PDH isoform 2 (G3PDH2, spot 3), aldosereductase (spot 4), ectodemal dysplasia receptor (EDAR) associated death receptor domain isoform CRA_a (EDARADD, spot 5), heat shock 60kD protein 1 variant 1 (HSP60, spot 8), heat shock 70kDa protein 5 precursor (HSP70, spot 11), 14-3-3 protein ζ/δ (spot 13), heat shock protein 90kDa beta member 1 precursor (HSP90, spot 15), Ran (spot 16) and ubiquitin activating enzyme E1 (UBE1, spot 17).

**Table 2:** PMF search results of the differently expressed proteins using Mascot search with NCBInrdatabases.

Spot (No.)	Protein ID	Accession No	MW (kDa)	pI	Trends in expression	Major Function
1	G3PDH isoform 1 [*Homo sapiens*]	gi|7669492	36.2	8.57	**↓**	Energy metabolism
3	G3PDH isoform 2 [*Homo sapiens*]	gi|378404908	31.7	7.15	**↓**	Energy metabolism
4	Aldosereductase [*Homo sapiens*]	gi|4502049	36.23	6.51	**↓**	Energy metabolism
5	EDAR-associated death domain, isoform CRA_a [*Homo sapiens*]	gi|119590453	42.66	5.64	**↑**	Apoptosis
8	heat shock 60kDa protein 1 variant 1 [*Homo sapiens*]	gi|189502784	60.81	5.83	**↑**	Molecular chaperones
11	heat shock 70 kDa protein **5** precursor [*Homo sapiens*]	gi|16507237	72.40	5.07	**↑**	Molecular chaperones
13	14-3-3 protein ζ/δ [*Homo sapiens*]	gi|49119653	30.1	4.72	**↑**	Signal transduction
15	heat shock protein 90kDa beta member 1 precursor [*Homo sapiens*]	gi|15010550	90.31	4.73	**↑**	Molecular chaperones
16	Ran [*Homo sapiens*]	gi|5453555	24.58	7.01	**↓**	Apoptosis
17	ubiquitin activating enzyme E1 [*Homo sapiens*]	gi|35830	118.807	5.5	**↑**	Protein degradation

To confirm the altered proteins identified by mass spectrometry, quantitative real-time RT-PCR assay was further applied. As shown in Fig 3B, after HMC stimulation for 48 h, *HSP60*, *HSP70*, *14-3-3 protein ζ/δ*, *HSP90*, *EDARADD* and *UBE1* mRNA expression were up-regulated about 1.16, 1.74, 1.46, 1.62, 2.2 and 1.7-fold, respectively, while the transcription of *Ran*, *G3PDH1*, *G3PDH2* and *aldosereductase* was decreased about 1.4, 1.9, 1.4 and 1.4-fold, respectively. These data were consistent with those of MALDI-TOF-TOF/MS analysis, suggesting these 10 proteins related to cell apoptosis, energy metabolism, molecular chaperones, or signal transduction, might be involved in the apoptosis of HeLa cells induced by HMC.

### Apoptosis was mediated by a mitochondria-initiated intrinsic pathway

**Fig 4:**
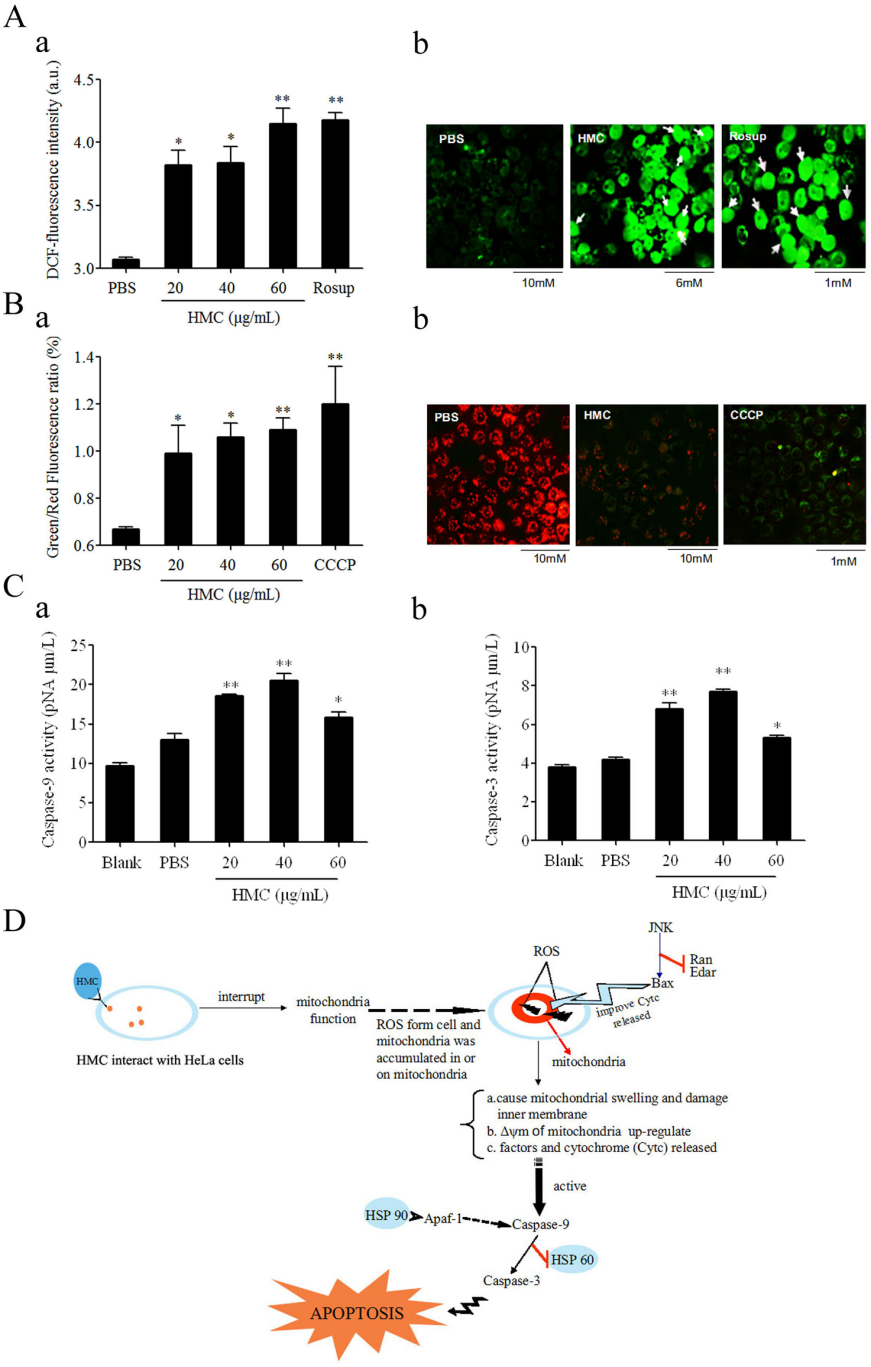
Molecular mechanism analysis of *Litopenaeus vannamei* HMC against HeLa cells. (A) Effects of HMC induced ROS generation. (a) Quantitative analysis of the fluorescence intensity of DCFH-DA in HeLa cells treated with HMC, PBS or Rosup for 48 h. (b) Micrographs show the corresponding DCFH-DA-fluorescence pictures of HeLa cells treated with PBS (1), HMC (2), and Rosup (3) (Magnification, 200×). The white arrows points to the fluorescence of ROS in HeLa cells. (B) HMC induced dissipation of Δψm in HeLa cells. (a) Quantitative analysis of the green/red fluorescence ration in HeLa cells treated with HMC, PBS or CCCP for 48 h. (b) Micrographs show that HeLa cells treated with PBS (1), HMC (2), and CCCP (3). (C) Measurement of caspase-9 (a) and caspase-3 (b) activities in HeLa cells treatment with HMC, and PBS for 48 h. Caspase-9/3 activities were represented with the concentration of pNA. The significance difference between the HMC- (or Rosup-, or CCCP-) and PBS-treated cells was indicated with one (*p* < 0.05) or two asterisks (*p* < 0.01). (D) Schematic diagram of the likely molecular mechanism of HMC against HeLa cells *in vitro*.

To test if the mitochondria-initiated pathway was involved in the HMC-induced apoptosis, the content of ROS in HeLa cells was measured. Incubation of HeLa cells with HMC at the concentration of 20–100 μg/mL for 48 h significantly (*p*<0.05 or 0.01) increased the intracellular level of ROS as compared to those in the control group of PBS (Fig 4A). Given that a high level of ROS could change mitochondrial permeability, we measured the membrane potential (Δψm) in mitochondria. As shown in Fig 4B, the ratio of green and red fluorescence increased significantly (*p*<0.05 or 0.01) in HeLa cells treated with HMC (20–100 μg/mL) for 48 h when compared to that of negative control group, suggesting the decrease of Δψm in cancer cells. Furthermore, we analyzed the activity of caspase-9/3, showing that the value in HeLa cells exposed to the different concentration of HMC (20–60 μg/mL) for 48 h were significantly (*p*<0.05 or 0.01) increased compared with those of negative control groups (Fig 4C). Therefore, these data suggested that HMC induced HeLa cells apoptosis might be involved in a mitochondria-initiated intrinsic pathway (Fig 4E).

## Discussion

It has been documented that cancers of the breast and cervix are the most common causes of cancer death worldwide for women, with cervical cancer the most common cause in 55 countries [38]. HeLa cell is the most widely used model cell line for studying human cellular and molecular biology [39, 40]. Evidence suggests that *Rapana thomasiana* HMC showed antiproliferative activity against human breast cancer MCF-7 and cervical cancer HeLa cells [41]. However, the antiproliferative effect of arthropod HMCs have not been reported. Here, we found for the first time HMC from *L*. *vannamei* could inhibit HeLa cells proliferation obviously (Fig 1). Further, morphological features, bound characterization between HMC and cells, and DNA fragmentation pattern (Fig 2) indicated that HeLa cells bound with HMC died of apoptosis.

Moreover, a total of 17 altered proteins were found from HeLa cells treated with HMC by comparative proteomics analysis (Fig 3A). Of these, 10 protein spots were successfully identified as G3PDH1, G3PDH2, aldosereductase, EDARADD, HSP60, HSP70, 14-3-3 protein ζ/δ, HSP90, Ran and UBE1 (Fig 3A and Table 2). In addition, the transcriptions of *HSP60*, *HSP70*, *14-3-3 protein ζ/δ*, *HSP90*, *EDARADD* and *UBE1* increased, while that of *Ran*, *G3PDH1*, *G3PDH2* and *aldosereductase* decreased significantly 48 h post HMC treatment (Fig 3B).

Evidence suggests that these differentially expressed proteins play a significant role in the induction of apoptosis in tumor cells [42–47]. For examples, silencing of Ran in various tumor cell types could cause aberrant mitotic spindle formation, mitochondrial dysfunction and apoptosis [43]. EDAR in MCF7 cells has been shown to be capable of activating the nuclear factor-κB, JNK, and caspase-independent cell death pathway [44]. Study by Chandra *et al*. [45] indicates that HSP60 has a pro-death function probably related to mitochondria-initiated caspase-dependent apoptosis. Liossis *et al*. [46] found that the over expression of HSP70 could enhance the TCR/CD3- and Fas/Apo-1/CD95-mediated apoptotic cell death in Jurkat T cells.

It has been documented that HMC is associated with ROS production in invertebrate [48, 12]. Coates *et al* found. [48] that HMC-derived phenoloxidase (PO) in amebocytes that is under pathologic condition to help them undergoing apoptosis induced by phagocytosis. Jiang *et al*. [12] reported that HMC from horseshoe crab was cleaved into PO, resulting in the production of ROS intermediate. Previous reports have indicated that ROS plays an important role in regulating and trigging mitochondria-initiated apoptosis [49–51]. Excessive amounts of ROS accumulated in mitochondrial could cause mitochondrial swelling and damage to the inner membrane [49, 50], subsequently, intermembrane proteins, such as apoptosis inducing factors and cytochrome (Cytc), would be released, and ultimately triggering the caspase cascade [51]. In this study, we showed clearly a significant increase of ROS concentration (Fig 4A), dissipation of Δψm (Fig 4B) and upregulation of caspase-9/3 activity (Fig 4C) in HeLa cells after 48 h treatment with HMC. These data indicated that mitochondria pathway was involved in the process of HMC-mediated HeLa cell apoptosis.

In summary, the present study showed that HMC from *L*. *vannamei* would be effective against HeLa cell growth *in vitro*. The likely molecular mechanisms underpinning the antiproliferative effect of HMC could be mitochondria mediated apoptosis pathway (Fig 4D). Further investigation will be required to explore the antitumor properties of *L*.*vannamei* HMC *in vivo* by use of the nude mice model.

## References

[1] van Holde KE, Miller KI (1995) Hemocyanins. Adv Protein Chem 47:1–81. 8561049 10.1016/s0065-3233(08)60545-8

[2] Adachi K, Wakamatsu K, Ito S, Miyamoto N, Kokubo T, Nishioka T, et al (2005) An oxygen transporter hemocyanin can act on the late pathway of melanin synthesis. Pigm Cell Res 18: 214–219. 10.1111/j.1600-0749.2005.00232.x 15892718

[3] Jaenicke E, Föll R, Decker H (1999) Spider hemocyanin binds ecdysone and 20-OH-ecdysone. J Biol Chem 274: 34267–34271. 10567401 10.1074/jbc.274.48.34267

[4] Paul RJ, Pirow R (1998) The physiological significance of respiratory proteins in invertebrates. Zoology 100: 298–306.

[5] Coates CJ, Nairn J (2014) Diverse immune functions of hemocyanins. Dev Comp Immunol 45: 43–55. 10.1016/j.dci.2014.01.021 24486681

[6] Decker H, Hellmann N, Jaenicke E, Lieb B, Meissner U, Markl J (2007) Minireview: Recent progress in hemocyanin research. Integr Comp Biol 47: 631–644. 10.1093/icb/icm063 21672868

[7] Nagai T, Osaki T, Kawabata SJ (2001) Functional conversion of hemocyanin to phenoloxidase by horseshoe crab antimicrobial peptides. J Biol Chem 276: 27166–27170. 11375396 10.1074/jbc.M102596200

[8] Jaenicke E, Fraune S, May S, Irmak P, Augustin R, Meesters C, et al (2009) Is activated hemocyanin instead of phenoloxidase involved in immune response in woodlice? Dev Comp Immunol 33:1055–1063. 10.1016/j.dci.2009.05.005 19447131

[9] Glazer L, Tom M, Weil S, Roth Z, Khalaila I, Mittelman B, et al (2013). Hemocyanin with phenoloxidase activity in the chitin matrix of the crayfish gastrolith. J Exp Biol 216: 1898–1904. 10.1242/jeb.080945 23393281

[10] Zhang X, Huang C, Qin Q (2004) Antiviral properties of hemocyanin isolated from shrimp *Penaeus monodon*. Antivir Res 61: 93–99. 14670582 10.1016/j.antiviral.2003.08.019

[11] Zanjani NT, Miranda-Saksena M, Valtchev P, Diefenbach RJ, Hueston L, Diefenbach E, et al (2016) Abalone hemocyanin blocks the entry of HSV-1 into cells: a potential new antiviral strategy. Antimicrob Agents Chemother 60: 1013–1012. 26643336 10.1128/AAC.01738-15 PMC4750698

[12] Jiang N, Tan CNS, Ho B, Ding JL (2007) Respiratory protein-generated active oxygen species as an antimicrobial strategy. Nat Immunol 8: 1114–1122. 17721536 10.1038/ni1501

[13] Destoumieux-Garzón D, Saulnier D, Garnier J, Jouffrey C, Bulet P, Bachère E (2001) Crustacean immunity: antifungal peptides are generated from the C terminus of shrimp hemocyanin in response to microbial challenge. J Biol Chem 276: 47070–47077. 11598107 10.1074/jbc.M103817200

[14] Guo D, Wang H, Zeng D, Li X, Fan X, Li Y (2011) Vaccine potential of hemocyanin from *Oncomelania hupensis* against *Schistosoma Japonicum*. Parasitol Int 60: 242–246. 10.1016/j.parint.2011.03.005 21440665

[15] Gesheva V, Idakieva K, Kerekov N, Nikolova K, Mihaylova N, Doumanova L, et al (2011) Marine gastropod hemocyanins as adjuvants of non-conjugated bacterial and viral proteins. Fish Shellfish Immunol 30: 135–142. 10.1016/j.fsi.2010.09.018 20887791

[16] Zhang YL, Wang SY, Peng XX (2004) Identification of a type of human IgG-like protein in shrimp *Penaeus vannamei* by mass spectrometry. J Exp Mar Biol Ecol 301: 39–54.

[17] Zhang YL, Wang SY, Xu A, Chen J, Lin B, Peng XX (2006) Affinity proteomic approach for identification of an IgA-like protein in *Litopenaeus vannamei* and study on its agglutination characterization. J Proteome Res 5: 815–821. 16602688 10.1021/pr0503984

[18] Zhang YL, Yan F, Hu Z, Zhao XL, Min SY, Du ZH, et al (2009) Hemocyanin from shrimp *Litopenaeus vannamei* shows hemolytic activity. Fish Shellfish Immunol 27: 330–335. 10.1016/j.fsi.2009.05.017 19501169

[19] Qiao J, Du ZH, Zhang YL, Du H, Guo LL, Zheng MQ, et al (2011) Proteomic identification of the related immune-enhancing proteins in shrimp *Litopenaeus vannamei* stimulated with vitamin C and Chinese herbs. Fish Shellfish Immunol 31: 736–745. 10.1016/j.fsi.2011.07.005 21767650

[20] Olsson CA, Chute R, Rao CN (1974) Immunologic reduction of bladder cancer recurrence rate. J Urol 111:173–176. 4810758 10.1016/s0022-5347(17)59919-x

[21] Riggs DR, Jackson BJ, Vona-Davis L, Nigam A, McFadden DW (2005) *In vitro* effects of keyhole limpet hemocyanin in breast and pancreatic cancer in regards to cell growth, cytokine production, and apoptosis. Am J Surg 189: 680–684. 15910720 10.1016/j.amjsurg.2004.10.005

[22] McFadden DW, Riggs DR, Jackson BJ, Vona-Davis L (2003) Keyhole limpet hemocyanin, a novel immune stimulant with promising anticancer activity in Barrett's esophageal adenocarcinoma. Am J Surg 186: 552–555. 14599624 10.1016/j.amjsurg.2003.08.002

[23] Arroyo JC, Gabilondo F, Llorente L, Meraz-Rios MA, Sanchez-Torres C (2004) Immune response induced *in vitro* by CD16- and CD16+ monocyte-derived dendritic cells in patients with metastatic renal cell carcinoma treated with dendritic cell vaccines. J Clin Immunol 24: 86–96. 14997038 10.1023/B:JOCI.0000018067.71622.fb

[24] Riggs DR, Jackson B, Vona-Davis L, McFadden D (2002) *In vitro* anticancer effects of a novel immunostimulant: keyhole limpet hemocyanin. J Surg Res 108: 279–284. 12505053 10.1006/jsre.2002.6548

[25] Antonova O, Dolashka P, Toncheva D, Rammensee HG, Floetenmeyer M, Stevanovic S (2014) *In vitro* antiproliferative effect of Helix aspersa hemocyanin on multiple malignant cell lines. Z Naturforsch C 69: 325–334. 25265853 10.5560/znc.2013-0148

[26] Gesheva V, Chausheva S, Mihaylova N, Manoylov I, Doumanova L, Idakieva K, et al (2014) Anti-cancer properties of gastropodan hemocyanins in murine model of colon carcinoma. BMC Immunol 15: 34 10.1186/s12865-014-0034-3 25168124 PMC4164791

[27] Arancibia S, Espinoza C, Salazar F, Del Campo M, Tampe R, Zhong TY, et al (2014) A novel immunomodulatory hemocyanin from the limpet *Fissurella latimarginata* promotes potentanti-tumor activity in melanoma. PLoS ONE 9: e87240 10.1371/journal.pone.0087240 24466345 PMC3900722

[28] Arancibia A, Campo MD, Nova E, Salazar F, Becker ML (2014) Enhanced structural stability of concholepas hemocyanin increases its immunogenicity and maintains its non-specific immunostimulatory effects. Eur J Immunol 42: 688–699. 10.1002/eji.201142011 22144228

[29] van Holde KE, Miller KI, Decker H (2001) Hemocyanins and invertebrate evolution. J Biol Chem 276: 15563–15566. 11279230 10.1074/jbc.R100010200

[30] Giomi F, Beltramini M (2007) The molecular heterogeneity of hemocyanin: its role in the adaptive plasticity of crustacea. Gene 398: 192–201. 17555892 10.1016/j.gene.2007.02.039

[31] Markl J (2013) Evolution of molluscan hemocyanin structures. Biochim Biophys Acta 1834: 1840–1852. 10.1016/j.bbapap.2013.02.020 23454609

[32] Sagias FG, Mitry RR, Hughes RD, Lehec SC, Patel AG, Rela M, et al (2010) N-acetylcysteine improves the viability of human hepatocytes isolated from severely steatotic donor liver tissue. Cell Transplant 19: 1487–1492. 10.3727/096368910X514620 20587150

[33] Shao ZJ, Zhang YY, Fan YY, Jin SJ, Yan J, Zheng XW, et al (2012) β, β- Dimethylacrylshikonin exerts antitumor activity via Notch-1 signaling pathway *in vitro* and *in vivo*. Biochem Pharmacol 15: 507–512. 10.1016/j.bcp.2012.05.013 22634048

[34] Murakawa M, Jung SK, Iijima K, Yonehara S (2001) Apoptosis inducing protein, AIP, from parasite-infected fish induces apoptosis in mammalian cells by two different molecular mechanisms. Cell Death Differ 8: 298–307. 11319613 10.1038/sj.cdd.4400811

[35] Cao JS, Wang ZH, Zhang YL, Qu FL, Guo LL, Zhong MQ, et al (2014) Identification and characterization of the related immune-enhancing proteins in crab *Scylla paramamosain* stimulated with rhubarb polysaccharides. Mol Immunol 57: 263–273. 10.1016/j.molimm.2013.10.003 24211534

[36] Pfaffl MW (2001) A new mathematical model for relative quantification in real-time RT-PCR. Nucleic Acids Res 29: e45 11328886 10.1093/nar/29.9.e45 PMC55695

[37] Bogdan C (2007) Oxidative burst without phagocytes: the role of respiratory proteins. Nat Immunol 8: 1029–1031. 17878909 10.1038/ni1007-1029

[38] Bray F, Jemal A, Grey N, Ferlay J, Forman D (2012) Global cancer transitions according to the Human Development Index (2008–2030): a population-based study. Lancet Oncol 13: 790–801. 10.1016/S1470-2045(12)70211-5 22658655

[39] Youn UJ, Fatima N, Chen QC, Chae S, Hung TM, Min BS. (2013) Apoptosis- inducing and antitumor activity of neolignans isolated from *Magnolia officinalis* in HeLa cancer cells. Phytother Res 27: 1419–1422. 10.1002/ptr.4893 23192855

[40] Lin MC, Lin SB, Chen JC, Hui CF, Chen JY (2010) Shrimp anti- lipopolysaccharide factor peptide enhances the antitumor activity of cisplatin *in vitro* and inhibits HeLa cells growth in nude mice. Peptides 31: 1019–1025. 10.1016/j.peptides.2010.02.023 20214941

[41] Guncheva M, Paunova K, Ossowicz P, Rozwadowski Z, Janus E, Idakieva K, et al (2015) Modification of *Rapana thomasiana* hemocyanin with choline amino acid salts significantly enhances its antiproliferative activity against MCF-7 human breast cancer cells. RSC Advs 5: 63345–63354.

[42] Wilde A, Zheng Y (2009) Ran out of the nucleus for apoptosis. Nat Cell Biol 11: 11–12. 10.1038/ncb0109-11 19122593

[43] Xia F, Lee CW, Altieri DC (2008) Tumor cell dependence on Ran-GTP directed. Cancer Res 68: 1826–1833. 10.1158/0008-5472.CAN-07-5279 18339863

[44] Kumar A, Eby MT, Sinha S, Jasmin A, Chaudhary PM (2001) The ectodermal dysplasia receptor activates the nuclear factor-kappaB, JNK, and cell death pathways and binds to ectodysplasin A. J Biol Chem 276: 2668–2677. 11035039 10.1074/jbc.M008356200

[45] Chandra D, Choy G, Tang DG (2007) Cytosolic accumulation of HSP60 during apoptosis with or without apparent mitochondrial release: evidence that its pro-apoptotic or pro-survival functions involve differential interactions with caspase-3. J Biol Chem 282: 31289–31301. 17823127 10.1074/jbc.M702777200

[46] Liossis SN, Ding XZ, Kiang JG, Tsokos GC (1997) Over expression of the heat shock protein 70 enhances the TCR/CD3- and Fas/Apo-1/CD95-mediated apoptotic cell death in Jurkat T cells. J Immunol 158: 5668–5675. 9190915

[47] Neckers L, Kern A, Tsutsumi S (2007) HSP90 inhibitors disrupt mitochondrial homeostasis in cancer cells. Chem Biol 14: 1204–1206. 18022558 10.1016/j.chembiol.2007.11.002

[48] Coates C, Whalley T, Wyman M, Nairn J (2013) A putative link between phagocytosis-induced apoptosis and hemocyanin-derived phenoloxidase activation. Apoptosis 18: 1319–1331. 10.1007/s10495-013-0891-x 23925540

[49] Cadenas E, Davies KJ (2000) Mitochondrial free radical generation, oxidative stress, and aging. Free Radical Bio Med 29: 222–230. 11035250 10.1016/s0891-5849(00)00317-8

[50] Liu C, Gong K, Mao X, Li W (2001) Tetrandrine induces apoptosis by activating reactive oxygen species and repressing Akt activity in human hepatocellular carcinoma. Int J Cancer 129:1519–1531. 10.1002/ijc.25817 21128229

[51] Dias N, Bailly C (2005) Drugs targeting mitochondrial functions to control tumor cell growth. Biochem Pharmacol 70: 1–12. 15907809 10.1016/j.bcp.2005.03.021

